# Comparative effect of anthocyanin on proliferation and migration of human gingival fibroblasts in the absence or presence of nicotine

**DOI:** 10.34172/japid.2023.018

**Published:** 2023-10-21

**Authors:** Sarina Azimian, Maryam Torshabi, Zeinab Rezaei Esfahrood

**Affiliations:** ^1^Department of Periodontics, Shahid Beheshti Dental School, Shahid Beheshti University of Medical Sciences, Tehran, Iran; ^2^Department of Dental Biomaterials, School of Dentistry, Shahid Beheshti University of Medical Sciences, Tehran, Iran; ^3^Department of Periodontics, School of Dentistry, Shahid Beheshti University of Medical Sciences, Tehran, Iran

**Keywords:** Anthocyanins, Fibroblasts, Migration, Nicotine, Proliferation

## Abstract

**Background.:**

Oral fibroblast malfunction can result in periodontal diseases. Nicotine can prolong the healing process as an irritant of oral tissues. Anthocyanins have been demonstrated to have potential benefits in preventing or treating smoking-related periodontal diseases. Cyanidin chloride’s (CC’s) potential in oral wound healing and the viability, proliferation, and migration of human gingival fibroblasts (HGFs) were examined in the presence and absence of nicotine by an in vitro study.

**Methods.:**

The effects of different nicotine concentrations (1, 2, 3, 4, and 5 mM) on the viability and proliferation of HGF cells were evaluated in the presence and absence of different CC concentrations (5, 10, 25, and 50 μM) using the quantitative MTT assay. The scratch test was performed to evaluate the migration of CC-treated cells in the presence of 2.5-mM nicotine.

**Results.:**

No cytotoxicity was observed at 1‒100 μM CC concentrations after 24, 48, and 72 hours of exposure to HGF cells. However, a concentration of 200 μM significantly reduced cell viability by about 20% at all the three-time intervals (*P*<0.05). Also, 3‒5-mM concentrations of nicotine significantly reduced cell viability in a dose- and time-dependent manner. Moreover, the understudied CC concentrations decreased nicotine’s adverse effects on cell migration to some extent.

**Conclusion.:**

Although the understudied CC concentrations could not significantly reduce the adverse effects of understudied nicotine concentrations on the viability and proliferation of HGF cells, they were able to reduce the detrimental effects of nicotine on cell migration significantly.

## Introduction

 Oral wound healing is a multi-phased process that includes cell proliferation and migration, collagen remodeling and deposition, wound contraction, and angiogenesis.^1,2^ Successful oral wound healing requires the complex coordination of various cell types, including fibroblasts, endothelial cells, macrophages, keratinocytes, and platelets.^2^ Human gingival fibroblasts (HGFs) are abundant cells in gingival connective tissue.^3^ Due to the role and importance of these cells, their damage results in gingival breakdown.^2^

 Several factors damage the oral mucosa, one of the most important of which is smoking. Smoking is known as an important risk factor for noncommunicable chronic diseases.^4^ Various in vitro studies have shown that nicotine and smoking can impair the proliferation, migration, and attachment of gingival fibroblasts.^5^

 Cigarette nicotine reduces oxygen supply to gingival tissues by decreasing gingival blood flow and the number of circulating cells.^6,7^ It reduces fibroblasts’ collagen and non-collagen protein production, negatively affecting oral fibroblasts’ viability, migration, and differentiation and causing periodontal tissue damage.^8-10^

 Inflammation and damage to the gingival and periodontal tissues occur due to the complex interactions between periodontal pathogens and immune system inflammatory responses. Among them, reactive oxygen species (ROS) and free radicals contribute significantly to the development and progression of periodontitis.^11^

 In some cases, periodontal treatment alone is not adequate. Although poor nutrition does not cause periodontal disease directly, many studies believe that disease progression is faster and more severe in people with poor diets due to an impaired host response.^12^

 Antioxidants are found in all body fluids and tissues. They constitute the body’s first line of defense against free radical damage caused by cigarette smoke, medications, illness, and stress.^13,14^ Recent studies have shown that antioxidant supplements are essential for preventing and successfully treating disorders of gingival tissue and other tooth-supporting structures.^8,15,16^

 Anthocyanins, a group of highly reactive antioxidants, can interact with free electrons in free radical molecules by donating an electron to oxygen species to reduce their toxic effects. Nicotine stimulates the production of free radicals, which is associated with oxidative stress and a deficient antioxidant defense mechanism. Therefore, anthocyanins may protect gingival tissues against nicotine toxicity by reducing free radicals.^17^

 Cyanidin chloride (CyCl or CC) suppresses the RANKL-stimulated nuclear factor-kappa B (NF-κB) signaling pathway and stimulates the nuclear factor erythroid 2-related factor 2 (Nrf2) signaling pathway. It should be noted that NF-κB and Nrf2 are the two main transcription factors involved in regulating oxidative reactions and cell proliferation.^18,19^

 In this in vitro study, we examined the antagonistic effects of anthocyanin and CC on the toxicity of nicotine to HGFs.

## Methods

###  Materials

 The HGF1 PI 1 (NCBI: C165) cell line used in this study was obtained from the Pasture Institute Cell Bank, Tehran, Iran. The anthocyanin CC, MTT (3- [4,5-dimethylthiazol-2-yl] -2,5-diphenyl tetrazolium bromide), DMSO (dimethyl sulfoxide), and crystal violet were purchased from Sigma-Aldrich (Germany). The nicotine was obtained from MP Biomedicals (France). DMEM (Dulbecco’s Modified Eagles Medium), FBS (Fetal Bovine Serum), and antibiotics were purchased from Gibco (UK).

###  Cell viability assay

 First, the cells were cultured with DMEM containing 10% fetal bovine serum (FBS) and 1% penicillin-streptomycin antibiotic (regular medium; RM). They were incubated at 37 °C under 5% CO_2_ and 95% humidity. The logarithmic phase cells were then treated with different concentrations of nicotine and anthocyanin CC (in RM) separately and in combination. Untreated cells (treated with RM alone; no cytotoxicity, 100% viability) were used as negative controls for cell viability, proliferation, and migration assays.

 In this study, we evaluated and compared nicotine’s cytotoxicity and its effect on the viability and proliferation of gingival fibroblasts in the presence or absence of the antioxidant anthocyanin, CC, by the MTT assay (ISO-10993-5; 2009). The nicotine concentration range used in this study was selected from earlier research.^20-22^ On the first day, the studied cells, which were in the logarithmic growth phase, were carefully counted and planted in each well of 96-well culture plates (3500 cells/100 µL of RM/well). The plates were then incubated in a cell culture incubator for 24 hours (37 °C, with 95% humidity and 5% CO_2_). On the second day of the study (50‒60% cell confluence), different concentrations of anthocyanin CC (1, 5, 10, 25, 50, 100, and 200 μM) were added to the wells separately (six identical replicates for each concentration). It should be noted that zero concentration of CC (under two conditions: RM alone and RM containing a concentration of DMSO found in the highest concentration of CC solution) was considered the control group (normal conditions of cell growth and proliferation). After 24 (for acute cytotoxicity), 48, and 72 (for chronic cytotoxicity) hours, the medium on the cells in each cell was gently and carefully drained and replaced with a culture medium (serum-free and antibiotic-free) containing 10% MTT. The plates were then incubated for 3 hours, and after confirming the formation of formazan crystals under an inverted microscope, MTT dye was drained from each well, and the same amount of DMSO solvent was added to each well. Then, the light absorption of the resulting color solutions was read using an ELISA reader. The average light absorption of each group treated with the investigated materials was divided by the average light absorption of the control group (untreated cells, non-treated, no cytotoxicity = 100% viable) and multiplied by 100 to determine the percentage of cell viability. According to the ISO-10-993-5 standard (2009), a group is considered cytotoxic if it reduces viability by more than 30% compared to the control group (viability rate falls below 70%).^23^

 After determining the selected nicotine concentrations (concentrations of 1, 2, 3, 4, and 5 mM) obtained from previous studies,^21,22^ and also selecting the appropriate concentrations of anthocyanin CC obtained from the MTT test (concentrations of 0, 5, 10, 25 and 50 μM), in the second phase of the study, the possible antagonistic effects of the studied antioxidant were investigated. Thus, HGFs (3500/well) were cultured in each well of 96-well cell culture plates on the first day at a logarithmic growth phase. The cells were then treated with different concentrations of nicotine in the presence of different concentrations of anthocyanins on the second day. It should be noted that the complete, RM alone, and without nicotine and CC, was considered as the control group. The MTT test was then performed to evaluate cell viability and proliferation percentages 24 and 72 hours after cell treatment.

###  Cell migration assay

 An in vitro scratch assay was performed to evaluate the HGF cell migration rate (which indicates the cell’s ability to repair) after stimulation with a selective concentration of nicotine (2.5 mM) and in the presence and absence of three selected concentrations (10, 25, and 50 mM) of the antioxidant anthocyanin CC.^22^

 On the first day of the study, 100 000 HGFs in the logarithmic growth phase were cultured in each well of a 24-well plate. On the second day, when the cells had reached 100% confluence, rapid and vertical scratches were made in each well using a 100-µL sterile tip (time zero). Each well was rinsed twice with RM to remove unattached cells. The cells were then treated with the indicated nicotine and CC concentrations. The cells treated with RM alone (without nicotine and CC) were considered the control group. At zero time and 24 hours after scratching, the medium in each well was drained, and the cells were washed twice with a cold (4 °C) positive PBS buffer (containing calcium and magnesium). The cells were fixed by adding 500 µL of 100% pre-cooled methanol to each well, and the mixture was allowed to remain at room temperature (RT) for 10 minutes. For staining, methanol was drained, and 0.5% crystal violet dye solution was added (10 minutes, RT). The dye solution was then drained, and the cells were washed three times with deionized water. A digital image of the cells at the scratch site was captured under an inverted light microscope at × 4 magnification. Finally, using the ImageJ software, the distance between the two edges of the scratches in the samples was quantified (in pixels), and the migration percentage was calculated.

###  Statistical analysis

 The results were statistically analyzed using GraphPad Prism 9 software and one-way ANOVA, followed by Tukey’s post hoc tests. A *P* value < 0.05 indicated statistically significant differences between the groups.

 Due to the laboratory nature of the research (in vitro), the use of cells in the cell culture environment did not have any special ethical considerations.

## Results

###  Effects of different CC concentrations on the viability and proliferation of HGFs

**Figure 1:**
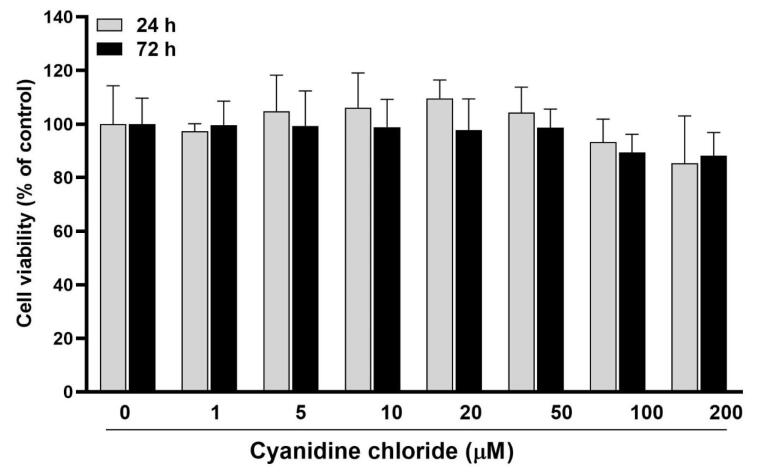
The effects of different anthocyanin CC concentrations (0 to 200 μM) on the viability and proliferation of HGFs 24, 48, and 72 hours after treatment

 At 24 and 72 hours after treatment (Figure 1), there were no statistically significant differences in cell viability and proliferation between 1‒100 μM concentrations of CC and also with the control group (zero CC concentration) (*P* > 0.05). At a 200-μM concentration, a 20% reduction in cell viability was observed in all three study time intervals (without significant differences between them). In general, none of the 1‒100 μM concentrations caused cytotoxicity (reduction of cell viability by > 30%). On the other hand, no significant proliferation (significant increase in the number of cells over time) was observed in any concentration. Therefore, four concentrations of 5, 10, 50, and 100 M were chosen for the subsequent investigation stage.

###  Effects of different nicotine concentrations on the morphology, viability, and proliferation of HGFs

**Figure 2:**
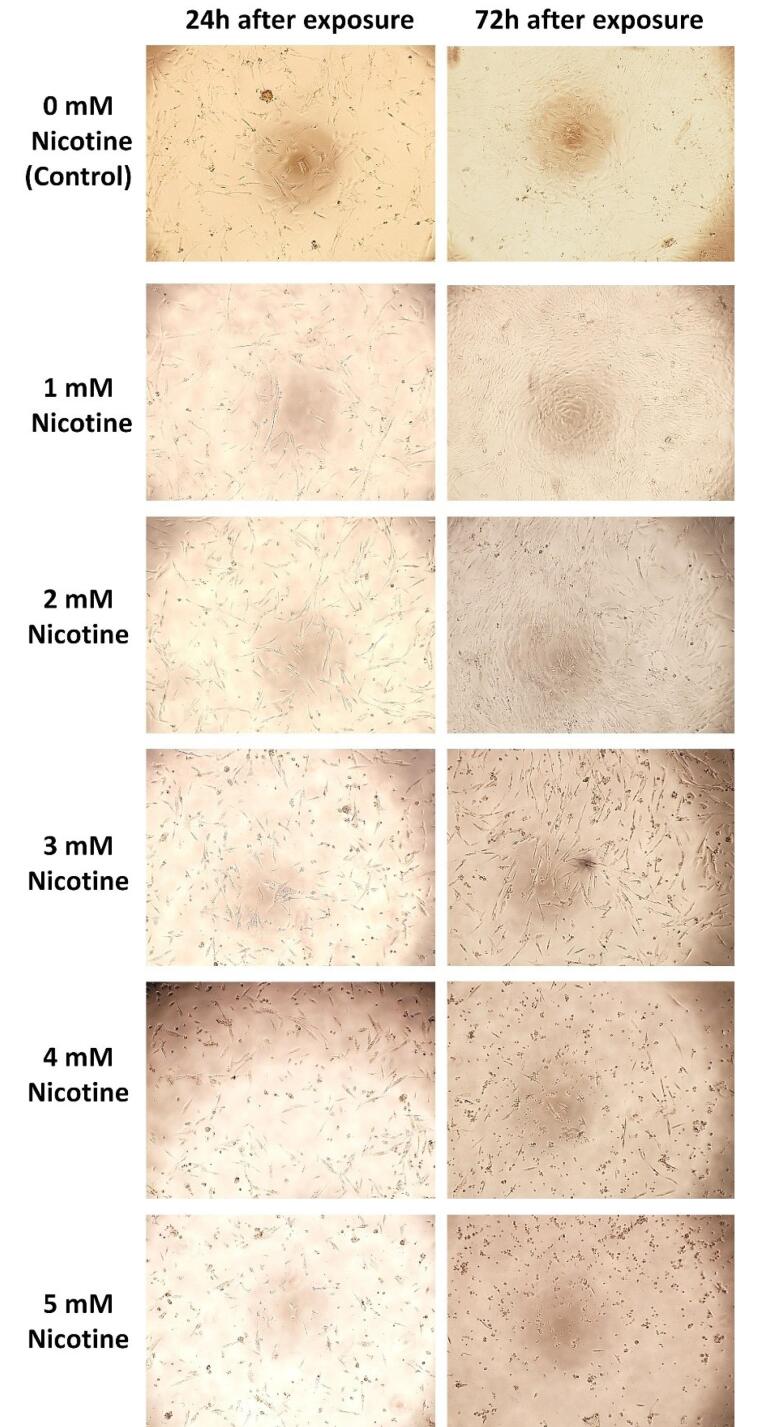
Qualitative (microscopic) observation of the morphology of HGFs exposed to zero (control) to 5-mM nicotine concentrations 24 and 72 hours after exposure

 A qualitative microscopic observation method ( × 10 magnification) was used to study the cell morphology. The morphology of HGF cells exposed to 1- and 5-mM concentrations of nicotine for 24 hours (acute toxicity) and 72 hours (chronic toxicity) was observed (Figure 2). At 1- and 2-mM concentrations of nicotine, the morphology was not very different from the control group (zero concentration) (no acute and chronic cytotoxicity). However, at 3- and 5-mM concentrations, especially 72 hours after exposure (chronic toxicity), a change in morphology was observed: vacuolization of cytoplasm, a decrease in the number of normal cells, and an increase in the number of apoptotic cells.

###  Effects of different nicotine concentrations on the viability and proliferation of HGFs in the presence and absence of different CC concentrations

**Figure 3:**
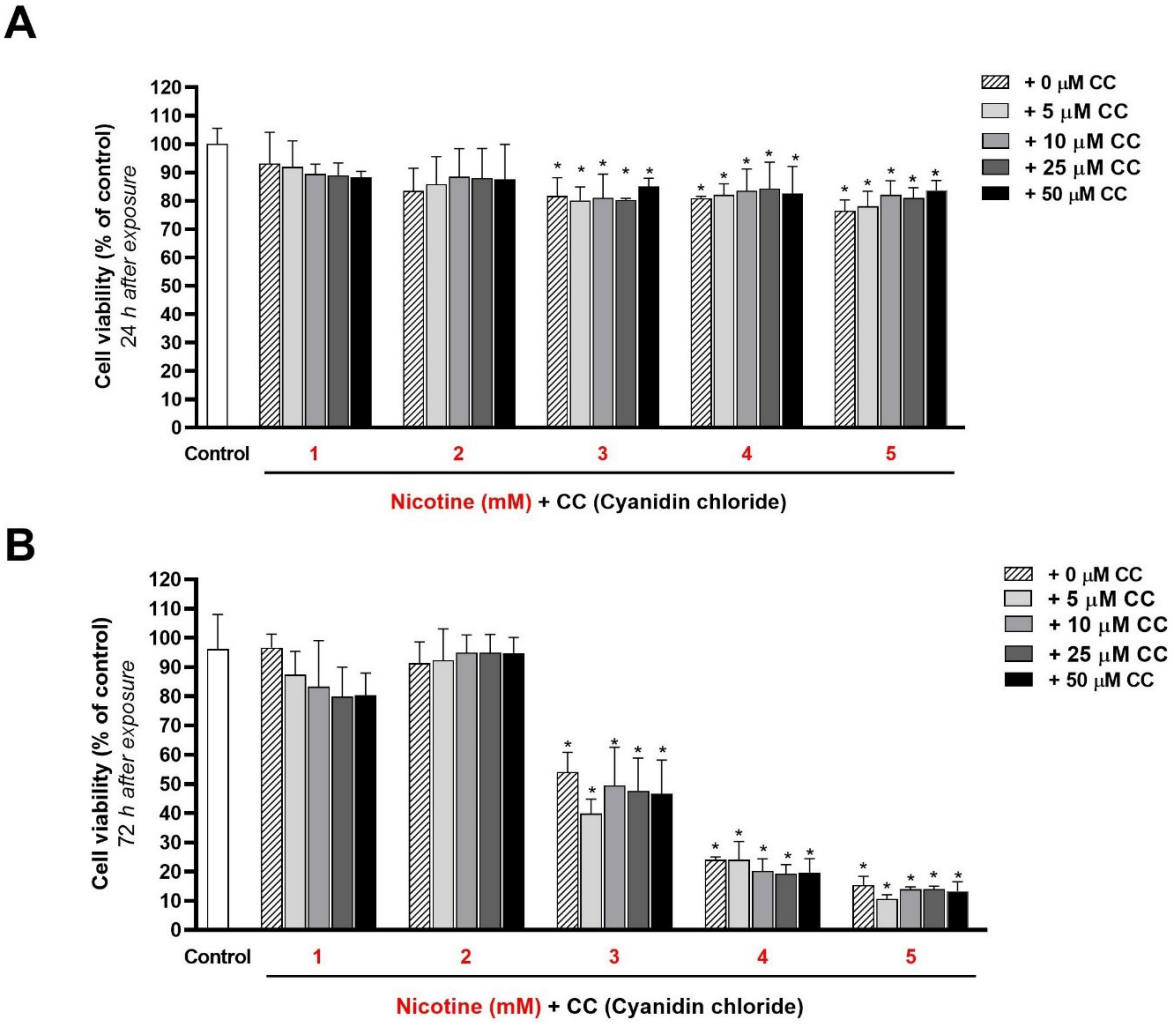
Quantitative evaluation (MTT test) of the effect of different concentrations of nicotine (1 to 5 mM) on the viability and proliferation of HGFs in the presence and absence of different concentrations (0 to 50 μM) of anthocyanin cyanidin chloride (CC), 24 (A) and 72 (B) hours after exposure. The stars on the columns show a statistically significant difference in the survival percentage of the target group compared to the control group (100% viability - zero concentration of nicotine and cyanidin chloride) (*P* < 0.05)

 After 24 hours of exposure (Figure 3A) (obtained from the results of the quantitative MTT test), a statistically significant decrease in cell viability percentage compared to the control group (zero concentration of nicotine) was observed at concentrations of 3, 4, and 5 mM (*P* < 0.05). However, the difference in viability percentages at 1- and 2-mM concentrations was not significant compared to the control group (*P* > 0.05).

 According to the results, adding CC at concentrations of 5, 10, 25, and 50 μM could not significantly change (increase or decrease) cell viability percentages in the cells exposed to all five nicotine concentrations (*P* < 0.05).

 After 72 hours of exposing HGFs to 1- and 2-mM concentrations of nicotine (Figure 3B) (from the results of the MTT quantitative test), no statistically significant increase or decrease in viability percentage was observed in the control group (*P* > 0.05). The results also showed that adding 5-, 10-, 25-, and 50-μM concentrations of CC to the cells stimulated with nicotine resulted in no statistically significant difference in cell viability.

 A dose-dependent decrease in cell viability was found with 3-, 4-, and 5-mM concentrations of nicotine 72 hours after exposure (30‒90% reduction in viability percentage compared to the control group). Adding 5-, 10-, 25-, and 50-μM concentrations of CC could not significantly change the cell viability percentage and reduce the adverse effects of nicotine.

###  Qualitative evaluation of the effect of nicotine on the HGFs’ migration in the presence and absence of different anthocyanin CC concentrations 

**Figure 4:**
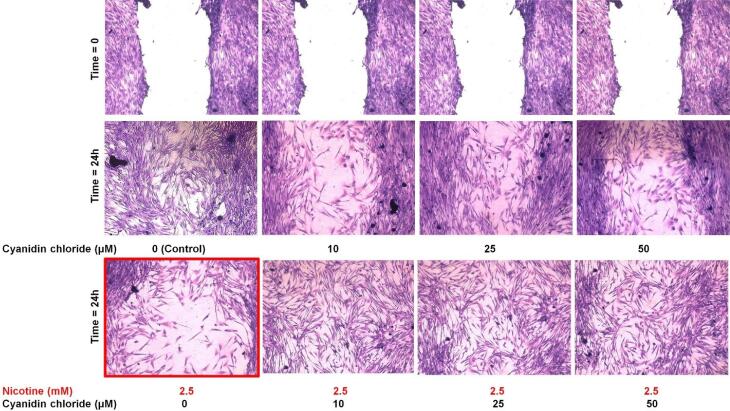
Qualitative rate of migration of HGF cells alone (control: without nicotine and cyanidin chloride), in the presence of nicotine alone (2.5 mM), cyanidin chloride alone (10, 25, and 50 mM), and nicotine with cyanidin chloride in zero time (the moment of scratching and exposure to the studied materials) and 24 hours after exposure. In the group exposed to 2.5-mM nicotine (without CC), the wound (space between the two edges of the scratch) opened more than (wider) zero time, and cytotoxicity was observed (red borderline)

 According to the results of MTT tests in the previous stages and primary cell migration tests, a 2.5-mM concentration of nicotine was selected for the final study of cell migration. Twenty-four hours after scratching and exposure to the studied materials, the wound in the control group (without nicotine and CC) was almost completely closed (Figure 4). The wound was not entirely closed after 24 hours at any of the three CC concentrations examined (without nicotine).

 In the group exposed to 2.5 mM of nicotine (without CC), the wound (space between the two edges of the scratch) opened more than (wider) zero time, and cytotoxicity was observed. However, cytotoxicity and reduced proliferation were seen more frequently in the nicotine-alone group than in the groups containing CC (without significant differences between concentrations).

## Discussion

 Today, we are witnessing an increase in smoking in different societies. Smoking is considered one of the most destructive risk factors for the development or progression of periodontal diseases. Tobacco smoke contains a variety of toxins, including nicotine (a highly addictive and fast-acting toxin), which affects many organs in the body. Cigarette nicotine significantly affects gingiva due to direct contact with epithelial cells and gingival fibroblasts. This substance prevents the adhesion and growth of gingival and periodontal ligament (PDL) fibroblasts.^8,20,24-30^

 In 1998, Alpar et al^31^ examined the toxic effects of 0.48‒62-mM nicotine on two PDLs and HGF cell lines. The results showed that the cytotoxicity of nicotine in the studied cells was dose-dependent, and especially 6-mM (973 μg/mL), 8-mM (1298 μg/mL), and 10-mM (1620 μg/mL) concentrations of nicotine reduced the viability. This study also showed that nicotine at concentrations > 3.9 mM caused cytoplasmic vacuolization and changes in the cytoskeleton’s vital components, such as microtubules and actin filaments (which play an essential role in cell structure and mitosis). These morphological changes were reversible at concentrations of 1.9 to 10.3 mM (by removing nicotine from the culture medium), but the damage was irreversible at higher doses.

 This in vitro study investigated the antioxidant potential of CC to reduce and antagonize the adverse biological effects of nicotine. According to the results of the cell viability and proliferation study by the MTT test, although the effect of this antioxidant in preventing the adverse biological effects of nicotine in the studied cells (HGF) was not significant, considerable effects were observed in the migration test and wound healing. However, due to the limitations of this laboratory study, confirmation of the initial cellular results requires more accurate cellular and molecular tests.

 In the present study, 3‒5-mM concentrations of nicotine after 24 hours of exposure induced vacuolization of the cytoplasm and a dose- and time-dependent decrease in cell viability and proliferation. The results related to the adverse biological effects of nicotine are consistent with previous studies.^31^

 This study showed that 2.5-mM nicotine reduced the density and migration rate of gingival fibroblasts, which can explain smokers’ slow wound healing rate. These results are consistent with studies that reported the adverse effects of smoking on wound healing. In 2005, a study showed that 16‒162 μg/mL of nicotine inhibited the HGF cell migration by acting on the Rac signaling pathway.^21^ In another study, HGFs were exposed to 0.025 to 32 μg/mL of nicotine, stimulating and inhibiting cell migration in low and high concentrations, respectively.^10^ Also, the results of a study in 2014 by Wheater and Mouabbi^32^ showed that nicotine at concentrations > 2 mM (400-400 μg/mL) was toxic to oral cells in a cell culture medium and reduced viability, adhesion, and migration of HGFs.

 Since cigarette nicotine disrupts the balance of the cell oxidant (destructive free radicals)‒antioxidant (anti-free radical) system, it leads to the weakening and inefficiency of the antioxidant system (natural cell defense).^14^ Investigating the antioxidant effect is of great interest in antagonizing and reducing the adverse biological effects of nicotine. Studies have shown that the antioxidant activity of polyphenols in plants has many positive effects on human health. For example, polyphenols, including anthocyanins, can neutralize free radicals and thus protect the tissue against many chronic diseases. Anthocyanins (from the flavonoid family) are potent antioxidants hat neutralize free radicals due to their high reactivity.^17^

 Few studies have investigated the antagonistic effect of antioxidants against nicotine.^21,22,33,34^ However, a comprehensive study on the antagonistic effect of the antioxidant anthocyanin CC against the toxic effects of nicotine has not been performed. In the present study, the toxic effects of different concentrations of nicotine, as well as the potential antagonistic effect of selected concentrations of CC on HGF cells, were investigated and compared.

 In 2016, Torshabi et al^21^ conducted an in vitro study to investigate the effect of nicotine in the presence and absence of vitamin E, a potent antioxidant, on the morphology, viability, proliferation, and expression of osteogenic genes in MG-63 osteoblast-like cells. In 2017, they conducted an in vitro study examining nicotine and cotinine effects on MG-63 osteoblast-like cells and HGF gingival fibroblasts in the presence and absence of two potent antioxidants: vitamins C and E. The results of the above studies showed that vitamin E (alone or in combination with vitamin C) at a concentration of 5 mM was more effective than vitamin C (at a concentration of 1 mM) in antagonizing the adverse biological effects of nicotine and its metabolite namely cotinine (in 5 mM concentration) on the viability, proliferation, migration, differentiation, and apoptosis of the understudied cells.^22^

 In 2010, San Miguel et al^33^ examined the effect of bioactive antioxidants (polyphenolic and turmeric derivatives at a concentration of 10^−5^ M of resveratrol (R), ferulic acid (F), phloretin (P), and tetrahydrocurcuminoids (T), [(RFT), (PFR), and (PF)]) on the proliferation and migration of human HGFs and periodontal ligament fibroblasts (HPDLs) pretreated with 6-mM nicotine for 2 hours. The results showed nicotine’s adverse biological effects on the viability and migration of the studied cells at concentrations > 2.5 mM, confirming the present study results. Also, they indicated that the cells treated with antioxidants showed more immigration rates than control cells and cells treated with nicotine. Double and especially triple combinations of these antioxidants (PFR and RFT) had more significant outcomes in counteracting nicotine effects and significantly increasing migration rates in HGF and HPDL than single antioxidants.^35^

 In 2021, Damrongrungruang et al^36^ conducted an in vitro study to investigate the effect of anthocyanin complex (AC) composed of cyanidin-rich extract and delphinidin-rich extract, and AC noisome gel on oral wound healing, examining cell viability, cell migration, nuclear morphology, and protein expression of HGFs. The results showed a significant increase in cell viability after treatment with 0.002, 0.02, and 0.2 mg/mL of AC niosomes and 0.02, 0.2, and 2 mg/mL of AC. Moreover, the cell migration was significantly increased after 24 hours of treatment with AC and AC noisome gels.

 In 2012, Desjardins et al^35^ showed that the anthocyanin cyanidin glucoside (at concentrations of 5 and 25 μg/mL) in combination with the other three anthocyanins (as black currant extract at concentrations of 5, 25, and 50 μg/mL) could significantly reduce the negative effect of nicotine on the viability of human gingival epithelial cells and fibroblasts at concentrations of 25 and 60 μg/mL of blackcurrant extract and 25 μg/mL of cyanidin glucoside. However, they did not examine cell migration.

 In contrast to the present study, which used only one type of anthocyanin, the mentioned study used several anthocyanins with different concentrations; anthocyanins may have synergistic effects when combined. Therefore, it turns out that the biological effects of the anthocyanin CC, compared to other anthocyanins, and their synergistic effects should be further investigated in future studies.

 In the present study, although different concentrations of CC alone (without nicotine) had no significant effect on the migration rate after 24 hours, significant effects were observed in improving migration (faster wound healing) in the presence of nicotine. As shown for the migration assay in Figure 4, nicotine alone adversely affected the migration of cells. The wound became worse and more open. However, the use of antioxidants led to healing of the wound and an increase in the number of cells.

 These findings were compatible with a 2009 study by Nizamutdinova et al,^37^ which showed that a 50-μM concentration of anthocyanins in soy did not cause significant cell migration in dermal fibroblasts. On the other hand, in 2018, Hoskin et al^38^ reported that the polyphenols in blueberries could induce the migration of human dermal fibroblasts after 24 hours. Therefore, discrepancies in the effects of anthocyanins on cell migration might be attributed to differences in anthocyanin type and the studied cell type.

 In 2011, San Miguel et al^34^ examined the effect of bioactive antioxidants (polyphenolic and turmeric derivatives at 10^−3^ to 10^−5^ M concentrations of resveratrol (R), ferulic acid (F), phloretin (P) and tetrahydrocurcuminoids (T); [(RFT), (PFR), and (PFT)]) on the proliferation and migration of HGFs and periodontal ligament fibroblasts (HPDLs). The results above showed that PFT increased cell migration; however, PFR and RFT did not significantly affect HGF wound healing rates.

 The anti-inflammatory potential of these antioxidants might be more significant than other biological effects. For example, in 2012 and 2013, Tipton et al^39,40^ reported that proanthocyanidins, cyanidin groups, and peonidin groups of anthocyanins in cranberry extract might inhibit IL-6 and IL-8 production in normal HGFs. They also showed that these substances could modulate inflammatory and proteolytic processes in HGFs and aggressive periodontitis.

 In contrast, a 2019 study by Jiang et al^41^ showed that CC (50 μM) could reduce the toxic effects of ZnO nanoparticles on colon cancer cells (CaCO_2_). However, this effect was not statistically significant. This substance could not significantly change the release rate of interleukin-8 induced by the above nanoparticles.

## Conclusion

 The present study showed that the adverse biological effects of nicotine on normal HGFs increased with increasing dose and time (dose-dependent and time-dependent toxicity). The antioxidants used in the present study could not reduce these adverse effects on cell viability and proliferation. However, anthocyanin and CC reduced the adverse effects of nicotine on cell migration to some extent. Due to the study’s limitations, additional comprehensive and cellular‒molecular research is necessary to evaluate this antioxidant’s beneficial biological effects. If the positive biological effects of the above antioxidant are proven, it can be used to prevent and treat oral diseases (gingival and periodontal tissues), especially in smokers.

## Competing Interests

 The authors state no conflict of interest.

## Data Availability Statement

 Data was presented in the manuscript.

## Ethical Approval

 The protocol of this study was approved by the Ethics Committee. (Ethical code: IR.SBMU.DRC.REC.1398.206).

## Funding

 The authors received no financial support for the publication of this article.
